# The impact of antithrombin III supplementation on prognosis during extracorporeal membrane oxygenation: a systematic review and meta-analysis

**DOI:** 10.1080/07853890.2025.2542439

**Published:** 2025-08-04

**Authors:** Ya-Ting Zeng, Yi-Nan Liu, Zi-Han Chen, Qiang Chen, Kai-Peng Sun

**Affiliations:** Department of Cardiac Surgery, Fujian Children’s Hospital (Fujian Branch of Shanghai Children’s Medical Center), College of Clinical Medicine for Obstetrics & Gynecology and Pediatrics, Fujian Medical University, Fuzhou, China

**Keywords:** ECMO, antithrombin III, mortality, bleeding and thrombotic complications

## Abstract

**Objective:**

To systematically evaluate the impact of antithrombin III (AT III) supplementation on the prognosis of patients undergoing extracorporeal membrane oxygenation (ECMO).

**Materials and methods:**

A comprehensive literature search was conducted in PubMed, Web of Science, Embase, and the Cochrane Library for studies assessing the effects of AT III supplementation on ECMO patient outcomes. The risk of bias was assessed using the Cochrane Risk of Bias and The Newcastle-Ottawa Scale.

**Results:**

A total of six studies involving 18,641 ECMO-treated patients were included. The meta-analysis showed that AT III supplementation did not reduce mortality in ECMO patients (RR = 1.17, 95% CI: 0.85–1.60, *p* = 0.34) and had no significant benefit in reducing bleeding events (RR = 1.04, 95% CI: 0.90–1.21, *p* = 0.56) or thrombosis (RR = 1.29, 95% CI: 0.81–2.05, *p* = 0.29). Subgroup analysis revealed that in cardiac ECMO patients, AT III supplementation was associated with an increased mortality but a reduced risk of thromboembolism. Conversely, in other ECMO support types, AT III supplementation was linked to a higher incidence of thromboembolism, with adult patients also showing an increased thromboembolism rate. No statistically significant differences were observed in other subgroup analyses.

**Conclusion:**

Overall, AT III supplementation does not reduce in-hospital mortality, bleeding, or thrombotic complications in ECMO patients and may even pose risks in certain populations. Therefore, routine AT III supplementation in ECMO patients may be not currently recommended.

## 1. Introduction

Extracorporeal membrane oxygenation (ECMO) is a mechanical circulatory and respiratory support system that facilitates gas exchange and hemodynamic stabilization through an external circuit. ECMO has been increasingly utilized for the management of refractory respiratory and cardiac failure, providing critical time for organ recovery or transplantation [1]. During ECMO, blood is drained from the patient’s venous system, passes through a centrifugal pump and oxygenator, and is then reinfused into the venous or arterial system. However, prolonged exposure to non-biological circuit surfaces activates the coagulation cascade, increasing the risk of thrombotic complications. As a result, systemic anticoagulation is required, but excessive anticoagulation raises the risk of life-threatening hemorrhage.

Unfractionated heparin (UFH) remains the most commonly used systemic anticoagulant in ECMO [2]. Heparin binds to antithrombin (AT), forming a complex that enhances the inhibition of multiple coagulation factors, including thrombin (factor IIa, factor Xa, factor XIIa, factor XIa, and factor IXa) [3,4]. However, up to 50% of ECMO patients experience heparin resistance, often due to acquired AT deficiency, which diminishes heparin’s anticoagulant effect [5,6]. This issue is particularly pronounced in pediatric patients, where acquired AT deficiency and heparin resistance are more frequent. Neonates are born with only 50% of adult AT levels, and preterm infants exhibit even lower AT activity. AT levels typically reach adult concentrations by 6–12 months of age [7,8]. In cases of heparin resistance, increasing heparin dosing fails to achieve expected anticoagulation targets, such as activated clotting time (ACT), activated partial thromboplastin time (APTT), or anti-Xa levels, resulting in either inadequate or excessive anticoagulation. In such scenarios, AT III supplementation has been proposed as a strategy to restore heparin responsiveness, optimize anticoagulation, and achieve therapeutic anticoagulation with lower heparin doses [9].

However, the safety and efficacy of AT III supplementation in ECMO remain uncertain, and routine AT III administration is still debated [10]. Therefore, this study aims to systematically evaluate and analyze the effects of AT III supplementation on in-hospital mortality, bleeding, and thrombotic complications in ECMO patients.

## 2. Materials and methods

### 2.1. Search strategy

A comprehensive literature search was conducted in PubMed, Web of Science, Embase, and the Cochrane Library to identify relevant studies examining the impact of AT III supplementation on ECMO patient prognosis. The search spanned from database inception until December 31, 2024. A combination of MeSH terms and free-text keywords was used, and the complete search strategy is detailed in Supplementary File 1. The primary keywords included ‘extracorporeal membrane oxygenation,’ ‘extracorporeal life support,’ ‘antithrombins,’ ‘antithrombin III,’ ‘serpin C1,’ ‘heparin cofactor I,’ and ‘kybernin.’ Additionally, a manual search was performed by reviewing the references of included studies. Two independent researchers screened the retrieved studies according to predefined inclusion and exclusion criteria, extracted data, and cross-checked the results. Any discrepancies were resolved through discussion with a third reviewer. This study was registered in PROSPERO (CRD42025649982).

### 2.2. Study selection criteria and data extraction

Inclusion criteria: (1) Studies involving ECMO patients; (2) Studies comparing AT III supplementation vs. non-supplementation groups; (3) Studies reporting complications such as bleeding, thrombosis, or in-hospital mortality during ECMO. Exclusion criteria: (1) Studies involving other forms of extracorporeal support (e.g. ventricular assist devices); (2) Studies where anticoagulants other than heparin (e.g. direct thrombin inhibitors) were used during ECMO; (3) Non-human studies, case reports, or case series.

Extracted data included: (1) Basic study information: title, first author, publication year, country, study design, data source, and sample size; (2) Patient characteristics: age, sex, ECMO type, ECMO duration, length of hospital stay, and primary disease; (3) Anticoagulation strategy: type and dosage of AT III, heparin dosage; (4) Outcomes: incidence of bleeding, thrombosis, and in-hospital mortality.

### 2.3. Study outcomes

Primary outcome: in-hospital mortality. Secondary outcomes: incidence of bleeding and thrombotic events.

### 2.4. Quality assessment

Two independent researchers assessed the risk of bias using the Cochrane Risk of Bias (RoB 2) tool for randomized controlled trials (RCTs) [11], evaluating bias across five domains: (1) Bias in the randomization process; (2) Bias due to deviations from intended interventions; (3) Bias from missing outcome data; (4) Bias in outcome measurement; (5) Bias in selective reporting. The Newcastle-Ottawa Scale (NOS) was used for observational studies, assessing: (1) Selection of study participants; (2) Comparability between groups; (3) Outcome assessment [12].

### 2.5. Statistical analysis

Meta-analysis was conducted using RevMan 5.3 and Stata 17.0. Binary variables were analyzed using risk ratios (RRs) with 95% confidence intervals (CIs). Heterogeneity was assessed using the *I*^2^ statistic: *I*^2^ ≤ 50% indicated low heterogeneity, and a fixed-effects model was used; *I*^2^ > 50% indicated high heterogeneity, and a random-effects model was used. Sensitivity and subgroup analyses were conducted to identify sources of heterogeneity. The results were visualized using forest plots, and publication bias was assessed using funnel plots and Egger’s test, with *p* < 0.05 considered statistically significant.

## 3. Results

### 3.1. Literature search results

**Figure 1.**
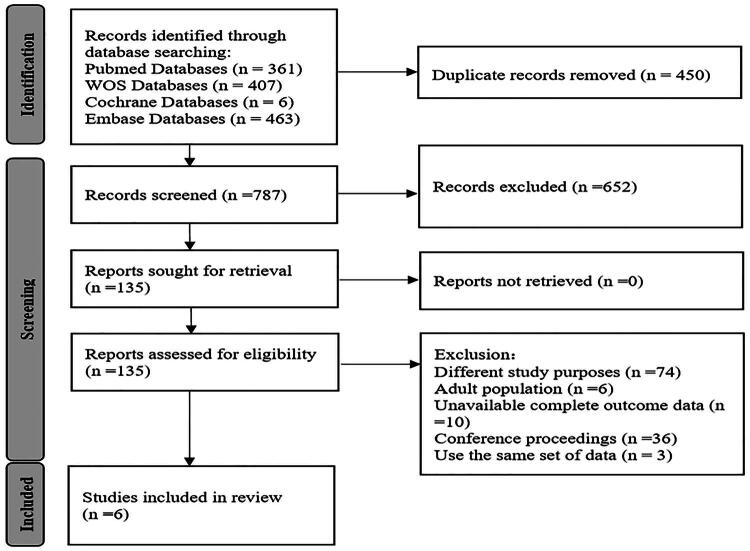
Study selection flowchart.

A total of 1237 articles were initially retrieved. After removing 450 duplicates, 787 articles remained. A total of 652 studies were excluded after title and abstract screening, and 129 studies were excluded after full-text review. Finally, six studies met the inclusion criteria and were included in the systematic review [13–18]. The selection process is illustrated in Figure 1.

### 3.2. Study characteristics and quality assessment

Among the included studies, one was an RCT, while five were retrospective cohort studies. Most participants were infants, primarily treated with human AT III concentrate, although no standardized AT III or heparin dosing protocol was consistently reported. The RCT was assessed as low risk of bias, while the five cohort studies had potential confounding limitations, with two studies identified as moderate risk of bias (see Supplementary File 2 for details). The characteristics of the included studies are summarized in Table 1.

**Table 1.** Characteristics of studies included in the meta-analysis.

Study	Study design	Multicenter study	Sample size	Age stratification	AT dose	AT type	indication of ECMO	bias risk
Aiello 2020	Cohort study	yes	9193	≤18 yr	NA	NA	Cardiac	Low
Colman 2019	Cohort study	no	123	>18 yr	NA	AT III	Other	Medium
Meshulami 2023	Cohort study	yes	514	≤18 yr	NA	AT III	Other	Medium
Panigada 2020	RCT	yes	48	>18 yr	2360 IU/d	AT III	respiratory	Low
Stansfield 2016	Cohort study	no	162	≤18 yr	125 IU/kg	AT III	respiratory	Low
Wong 2016	Cohort study	yes	8601	≤18 yr	NA	AT III	Other	Low

AT: antithrombin; ECMO: extracorporeal membrane oxygenation; RCT: randomized controlled trial.

### 3.3. Meta-analysis results

#### 3.3.1. Effect of AT III supplementation on mortality

**Figure 2.**
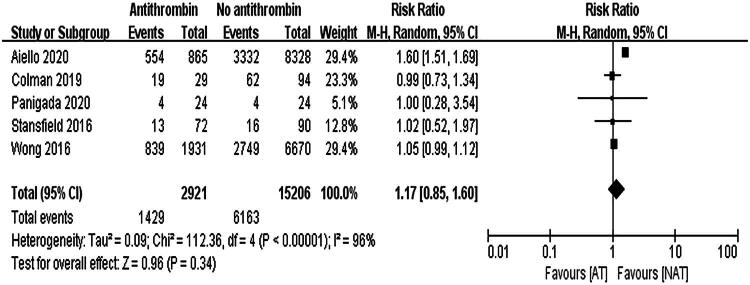
Forest Plot depicting mortality risk.

Among the six included studies, one study did not report mortality data and was therefore excluded from the mortality meta-analysis [15]. The remaining five studies included 18,127 ECMO patients, of whom 2,921 received AT III supplementation (mortality rate 48.9%) and 15,206 did not receive AT III (mortality rate 40.5%). Due to high heterogeneity (*I*^2^ = 96%, *p* < 0.001), a random-effects model was used, revealing no significant difference in mortality between the two groups (RR = 1.17, 95% CI: 0.85–1.60, *p* = 0.34). The results are shown in Figure 2.

#### 3.3.2. Effect of AT III on bleeding complications

**Figure 3.**
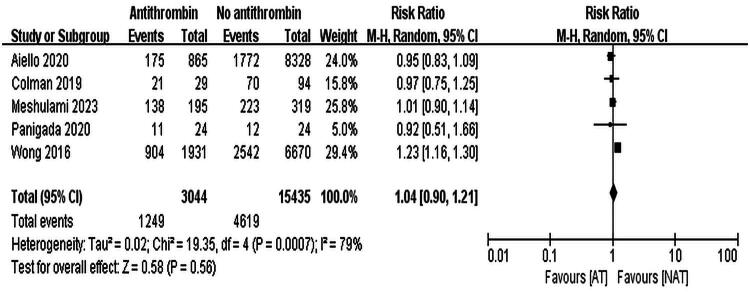
Forest Plot showing the incidence of hemorrhagic events.

Five studies reported bleeding complications [13–17]. A total of 18,479 ECMO patients were included, with 3,044 in the AT III group (bleeding rate 41.0%) and 15,435 in the non-AT III group (bleeding rate 29.9%). Due to high heterogeneity (*I*^2^ = 79%, *p* < 0.001), a random-effects model was used, and the results showed no significant difference in bleeding risk (RR = 1.04, 95% CI: 0.90–1.21, *p* = 0.56). The results are shown in Figure 3.

#### 3.3.3. Effect of AT III on thrombotic events

**Figure 4.**
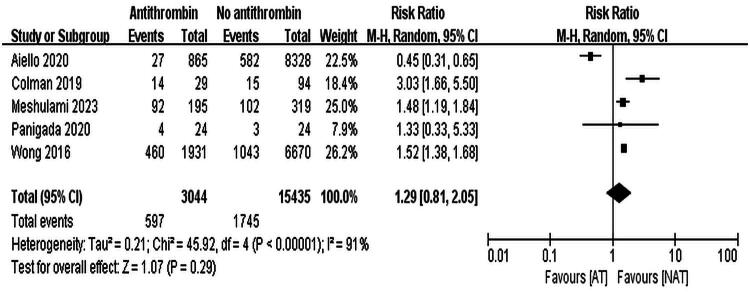
Forest Plot displaying the incidence of thrombotic events.

Five studies reported thrombotic events [13–17]. A total of 18,479 ECMO patients were included, with 3,044 in the AT III group (thrombosis rate 19.6%) and 15,435 in the non-AT III group (thrombosis rate 11.3%). Due to high heterogeneity (*I*^2^ = 91%, *p* < 0.001), a random-effects model was used, and the results showed no significant difference in thrombosis risk (RR = 1.29, 95% CI: 0.81–2.05, *p* = 0.29). The results are shown in Figure 4.

#### 3.3.4. Sensitivity analysis

**Figure 5.**
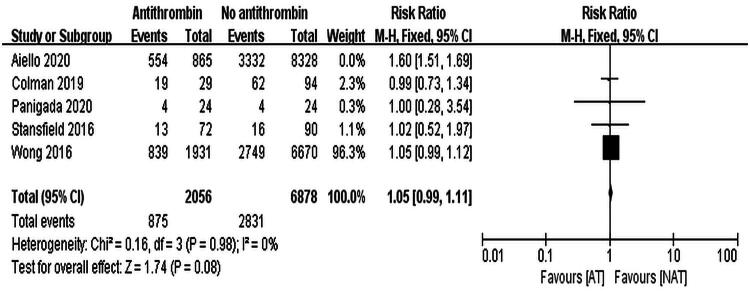
Forest Plot of mortality, reanalyzed using a fixed-effects model after removing one study with high heterogeneity.

Sensitivity analysis was performed for mortality, bleeding, and thrombosis. Aiello et al. significantly contributed to mortality heterogeneity [16]. Excluding this study reduced heterogeneity (*I*^2^ = 0%), and reanalysis using a fixed-effects model confirmed no significant mortality difference (RR = 1.05, 95% CI: 0.99–1.11, *p* = 0.08), as shown in Figure 5.

**Figure 6.**
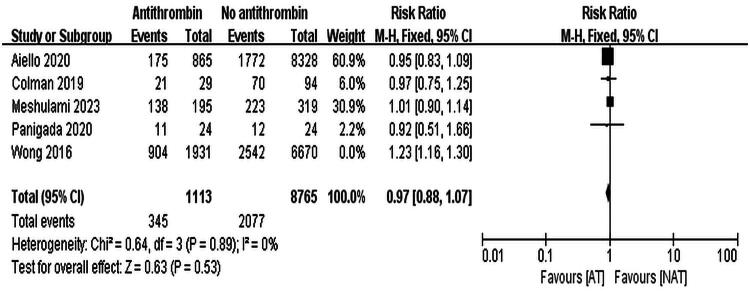
Forest Plot of hemorrhage incidence, reanalyzed using a fixed-effects model after removing one study with high heterogeneity.

Wong et al. significantly contributed to the heterogeneity in bleeding incidence [14]. Excluding this study reduced heterogeneity (*I*^2^ = 0%), and reanalysis using a fixed-effects model confirmed no significant difference in the incidence of bleeding events (RR = 0.97, 95% CI: 0.88–1.07, *p* = 0.53), as shown in Figure 6.

**Figure 7.**
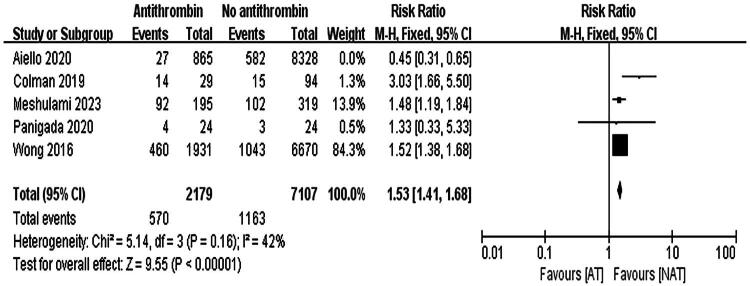
Forest Plot of thrombosis incidence, reanalyzed using a fixed-effects model after removing one study with high heterogeneity.

Aiello et al. substantially contributed to the heterogeneity in thrombosis incidence [16]. Excluding this study reduced heterogeneity (*I*^2^ = 42%), and reanalysis using a fixed-effects model indicated that patients receiving AT III had a higher risk of thrombosis events (RR = 1.53, 95% CI: 1.41–1.68, *p* < 0.001), as shown in Figure 7.

#### 3.3.5. Subgroup analysis

**Figure 8.**
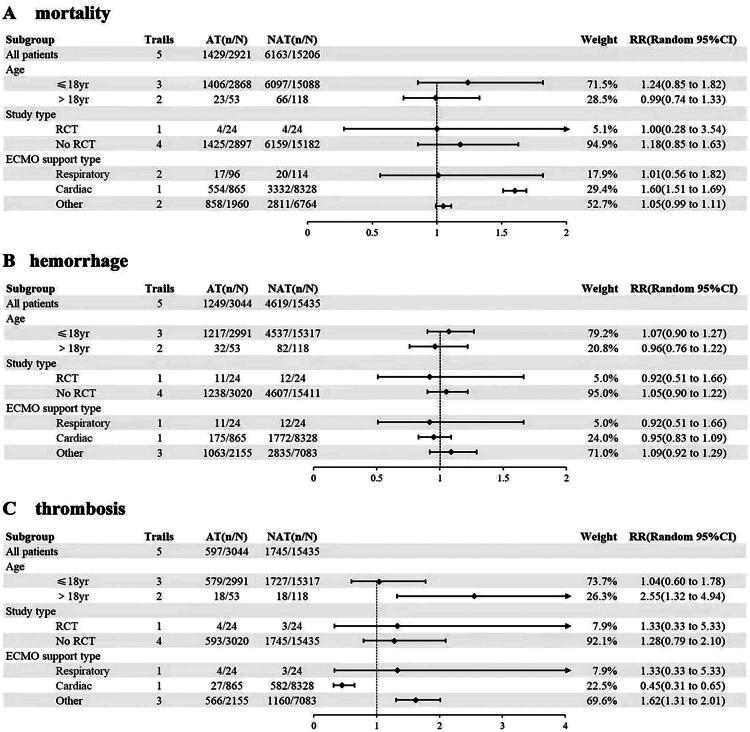
(A) Forest Plot of mortality subgroup analysis based on age, study type and ECMO support type. (B) Forest plot of subgroup analysis of hemorrhage events. (C) Forest plot of subgroup analysis of thrombotic events.

To further identify the sources of heterogeneity, subgroup analyses were performed on mortality, bleeding incidence, and thrombus occurrence. Based on patient age (>18 years, ≤18 years), study type (RCT, non-RCT), and type of ECMO support (respiratory, cardiac, other), 3 subgroups were then established. The results showed that in cardiac support ECMO, patients receiving AT III treatment had increased mortality (RR = 1.60, 95% CI: 1.51–1.69, *p* < 0.001), while there was no significant difference in mortality between the AT III group and the non-AT III group in respiratory support (RR = 1.01, 95% CI: 0.56–1.82, *p* = 0.97) and other support types (RR = 1.05, 95% CI: 0.99–1.11, *p* = 0.08). Age and study type did not show significant differences in mortality between the two groups (Figure 8A). There were no significant differences in bleeding incidence across the subgroups (Figure 8B). Adult patients receiving AT III treatment had a higher risk of thrombosis (RR = 2.55, 95% CI: 1.32–4.94, *p* = 0.005), while there was no significant difference in thrombotic complications in pediatric patients (RR = 1.04, 95% CI: 0.60–1.78, *p* = 0.90). In cardiac ECMO, the thrombosis rate was lower in the AT III group (RR = 0.45, 95% CI: 0.31–0.65, *p* < 0.001), whereas in other support types ECMO, the thrombosis risk was higher in the AT III group (RR = 1.62, 95% CI: 1.31–2.01, *p* < 0.001). There was no significant difference in thrombotic complications between the two groups in respiratory support ECMO (RR = 1.33, 95% CI: 0.33–5.33, *p* = 0.68). There were no significant differences in thrombosis rates between the two groups based on study type (Figure 8C).

#### 3.3.6. Publication bias

Funnel plots were constructed based on mortality, bleeding, and thrombotic event rates to assess publication bias in the pooled results. Visual inspection revealed approximate symmetry of the funnel plots (Supplementary File 3). Egger’s test demonstrated no significant publication bias (*p* = 0.782; *p* = 0.140; *p* = 0.672).

## 4. Discussion

This systematic review and meta-analysis indicate that AT III supplementation does not reduce mortality in ECMO patients and has no significant beneficial effect on the incidence of bleeding or thrombotic complications. To better evaluate the safety and efficacy of AT III supplementation and explore sources of heterogeneity, further subgroup analysis revealed that among different ECMO support types, only cardiac ECMO patients exhibited a significant difference in in-hospital mortality between the AT III and non-AT III groups (RR = 1.60, 95% CI: 1.51–1.69). Age and study type did not show significant differences in mortality between groups. Regarding thrombotic complications, AT III supplementation was associated with a reduced risk of thromboembolism in cardiac ECMO patients (RR = 0.45, 95% CI: 0.31–0.65) but was linked to an increased thromboembolism risk in adult patients (RR = 2.55, 95% CI: 1.32–4.94) and in other ECMO support types (RR = 1.62, 95% CI: 1.31–2.01). Study type did not show a significant difference in thrombotic complications between groups. Bleeding incidence did not significantly differ across subgroups based on age, study type, or ECMO support type.

This meta-analysis found no statistically significant difference in mortality between the AT III and non-AT III groups among ECMO patients. However, subgroup analysis suggested that AT III may increase mortality risk in cardiac ECMO patients. This finding is primarily based on a single large retrospective multicenter study involving 9,193 patients [16]. While the large sample size lends a certain degree of reliability to this finding, the reliance on one study limits the generalizability and certainty of the conclusion. Further mechanistic studies are warranted to explore the underlying reasons for this observed association.

Aiello et al. reported that AT III use was associated with an increased in-hospital mortality and reduced length of hospital stay in pediatric patients [16]. This may be influenced by institutional variations in medication protocols and differences in disease severity. Critically ill patients commonly present with sepsis, acidosis, massive transfusion, and dilution from large volumes of crystalloid infusion, all of which contribute to disseminated intravascular coagulation and acquired AT deficiency [19]. Under an AT III-targeted supplementation strategy, critically ill patients are more likely to receive AT III replacement therapy [9]. A separate study on perioperative AT III supplementation during cardiopulmonary bypass (CPB) also reported an increased in-hospital mortality rate with AT III use [20]. In contrast, observational studies in critically ill patients have found that AT III supplementation does not increase mortality [21,22]. Indeed, it is important to note that this elevated mortality may be attributed to the severity of pre-existing conditions rather than a causal effect of AT III supplementation [23]. The relationship between AT III supplementation and mortality risk factors was not explicitly addressed in these studies, and further control for such confounders is needed. Based on current findings, the use of AT III in cardiac ECMO patients should be approached with caution.

The interaction between AT III and heparin is fundamental to coagulation regulation, as AT III inhibits thrombin generation and fibrin formation, thereby preventing clot formation [24]. A commonly cited concern regarding AT III supplementation is the increased risk of bleeding, which can be life-threatening in critically ill patients. However, our study found that AT III supplementation did not increase the risk of bleeding in ECMO patients. Some studies suggest that in patients with low AT III activity and low anti-Xa levels, AT III supplementation restores heparin responsiveness without affecting heparin dosing or bleeding risk [25,26]. Similarly, a study on perioperative CPB found that AT III administration did not increase bleeding risk [20]. Higher doses of AT III may allow for lower heparin doses to achieve anticoagulation goals without increasing bleeding risk [9,27]. However, heparin responsiveness is not solely determined by AT III levels [28], and clinical AT III supplementation is often empirically guided, with no current studies quantitatively defining the threshold at which AT III-heparin complexes lead to increased bleeding risk.

Although the meta-analysis found no statistically significant difference in thromboembolism incidence between the AT III and non-AT III groups, there was substantial heterogeneity across studies. Aiello et al. reported that AT III use was associated with lower thrombosis incidence but higher in-hospital mortality, which may be related to early mortality in critically ill patients and shorter ECMO durations, making it difficult to accurately estimate AT III’s true efficacy [16]. Additionally, adult ECMO patients receiving AT III had a higher risk of thrombotic events, which contradicts the expected anticoagulant effects of AT III, raising the possibility of a false-positive result due to limited sample size. Meanwhile, some reports suggest that AT III products may inadvertently promote thrombosis. During the manufacturing process, latent antithrombin (LAT), a hyperstable procoagulant variant of AT III, may be present, which has been associated with severe thrombosis in critically ill patients [29,30]. In retrospective studies, a more plausible explanation is confounding bias, where patients with severe illness, heparin resistance, or known thrombotic conditions are more likely to receive AT III therapy. In contrast, RCTs evaluating AT III supplementation found no significant difference in thromboembolism rates between the AT III and control groups [20]. Furthermore, in neonates receiving ECMO, AT III supplementation was associated with a reduction in thrombotic events [18]. Therefore, although AT III’s role in reducing thrombosis remains unclear, there is no strong evidence suggesting AT III concentrates promote thrombosis.

Currently, no established guidelines define the optimal dose, timing, or frequency of AT III supplementation in ECMO. Most ECMO centers consider AT III supplementation based on target ACT levels, leading to significant variability in practice [31]. The heterogeneity in AT III dosing protocols across centers significantly undermines the comparability of studies and complicates the interpretation of existing evidence [10]. To address this, there is an urgent need for future prospective trials to adopt uniform thresholds for AT III supplementation to enhance the consistency and generalizability of findings. No consistent data indicate that AT III monitoring or supplementation improves outcomes, and more evidence is needed before recommending routine AT III use in ECMO [2]. Previous studies have remained inconclusive regarding the efficacy and safety of AT III. Therefore, we strongly advocate for prospective, well-designed randomized controlled trials stratified by patient age, ECMO type, and baseline AT levels to clarify the efficacy and safety of AT III supplementation and inform evidence-based clinical practice.

This study has several limitations. First, most included studies were retrospective, with limited RCT data, leading to potential confounding factors. Variations in AT III dosage and administration duration further complicate the interpretation of patient outcomes. Second, ECMO studies are limited by long case collection periods and small sample sizes, particularly in adult ECMO populations, affecting statistical power and representativeness. Third, some studies lacked mortality or complication data, leading to potential information loss. Conference abstracts were also reviewed, but full-text availability and data extraction limitations prevented their inclusion in the meta-analysis.

## 5. Conclusion

This study demonstrates that AT III supplementation does not reduce in-hospital mortality, bleeding, or thrombotic event rates in ECMO patients. Moreover, AT III may increase mortality and thrombotic risk in certain populations. Therefore, in the absence of compelling evidence of benefit and given the potential risks, routine AT III supplementation cannot be recommended outside of well-defined, protocolized, and closely monitored clinical settings.

## Supplementary Material

PRISMA checklist.docx

Supplementary File 1.tif

Supplementary File 3A.tif

Supplementary File 2B.tif

Supplementary File 3C.tif

Supplementary File 2A.tif

Supplementary File 3B.tif

## Data Availability

The datasets used and/or analysed during the current study are available from the corresponding author on reasonable request.
