# Supplementary figures and images for: The impact of antithrombin III supplementation on prognosis during extracorporeal membrane oxygenation: a systematic review and meta-analysis
Source: Ann Med. 2025 Aug 4;57(1):2542439. doi: 10.1080/07853890.2025.2542439 (PMC12322988; doi:10.1080/07853890.2025.2542439)

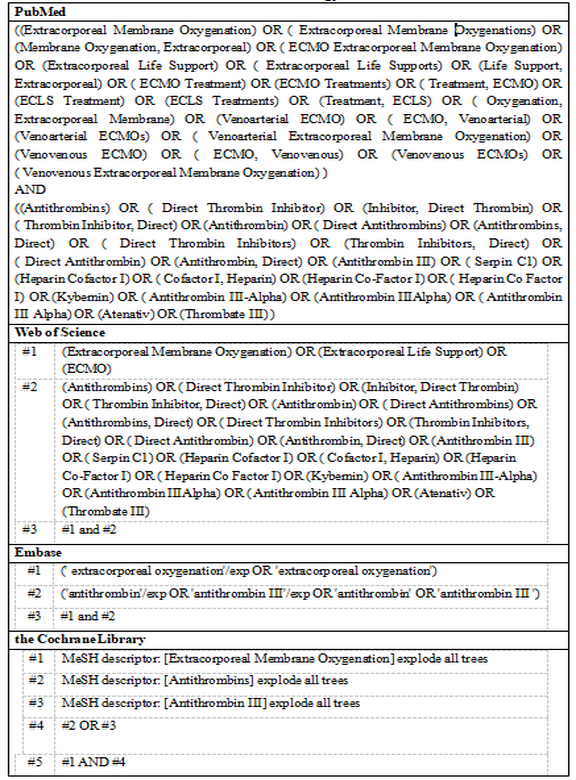

Supplement: Supplementary File 1.tif [file IANN_A_2542439_SM8279.tif]

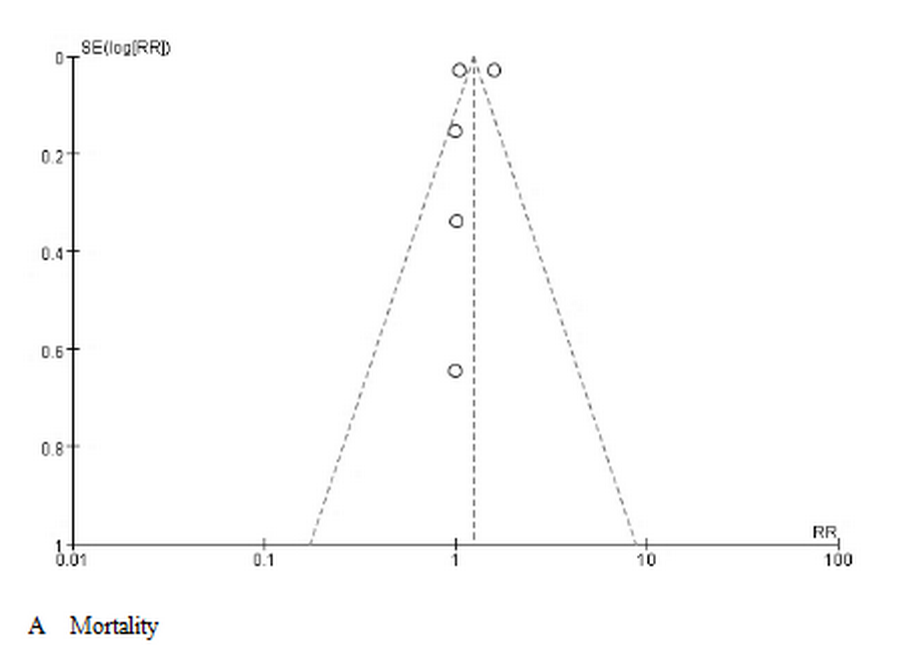

Supplement: Supplementary File 3A.tif [file IANN_A_2542439_SM8278.tif]

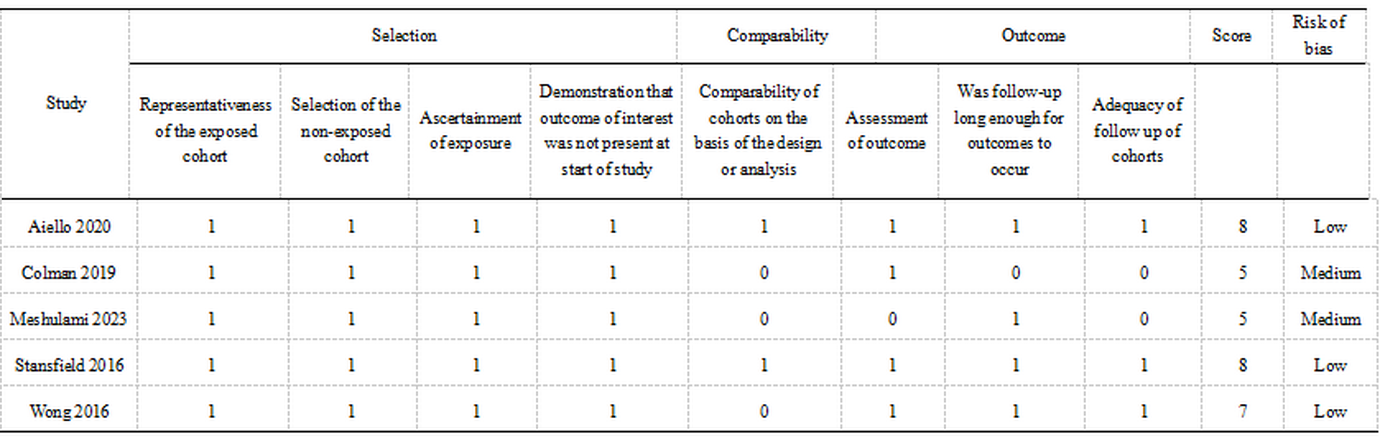

Supplement: Supplementary File 2B.tif [file IANN_A_2542439_SM8277.tif]

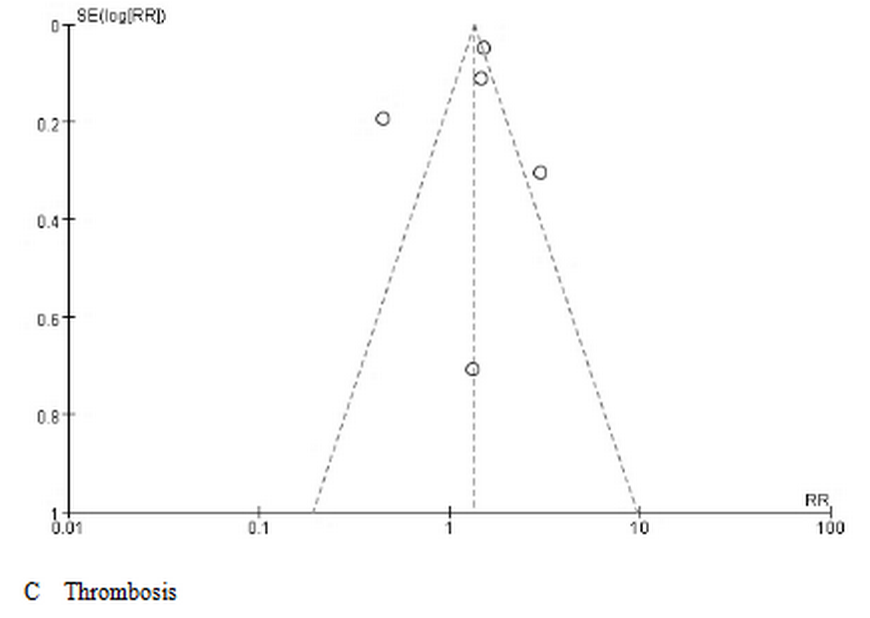

Supplement: Supplementary File 3C.tif [file IANN_A_2542439_SM8276.tif]

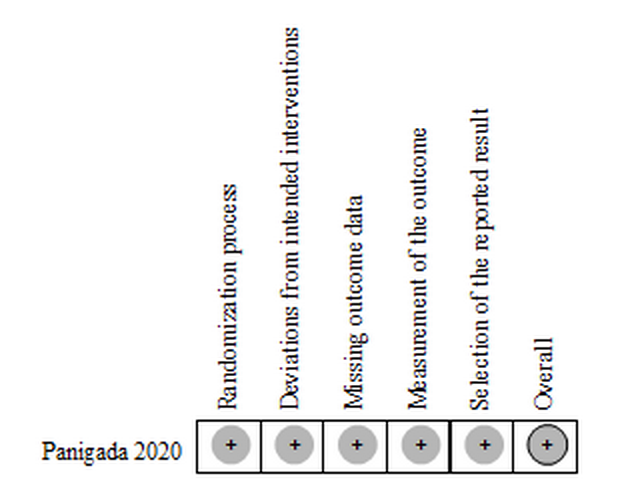

Supplement: Supplementary File 2A.tif [file IANN_A_2542439_SM8275.tif]

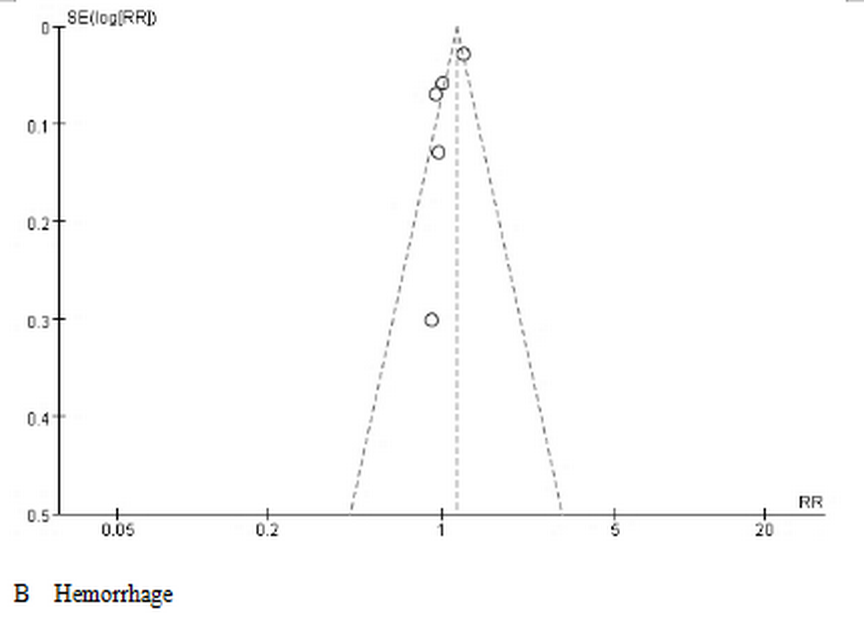

Supplement: Supplementary File 3B.tif [file IANN_A_2542439_SM8274.tif]
